# Assessment of fecal calprotectin and fecal occult blood as point-of-care markers for soil-transmitted helminth attributable intestinal morbidity in a case-control substudy conducted in Côte d'Ivoire, Lao PDR and Pemba Island, Tanzania

**DOI:** 10.1016/j.eclinm.2021.100724

**Published:** 2021-01-30

**Authors:** Chandni Patel, Ladina Keller, Sophie Welsche, Jan Hattendorf, Somphou Sayasone, Said M. Ali, Shaali M. Ame, Jean Tenena Coulibaly, Eveline Hürlimann, Jennifer Keiser

**Affiliations:** a Medical Parasitology and Infection Biology, Swiss Tropical and Public Health Institute, Basel, Switzerland; b University of Basel, Basel, Switzerland; c Department of International Program for Health in the Tropics, Lao Tropical and Public Health Institute, Vientiane, Lao People's Democratic Republic; d Public Health Laboratory Ivo de Carneri, Chake Chake, Pemba, Zanzibar, Tanzania; e Unité de Formation et de Recherche Biosciences, Université Félix Houphouët-Boigny, Abidjan, Côte d'Ivoire; f Department of Research and Development, Centre Suisse de Recherches Scientifiques en Côte d'Ivoire, Abidjan, Côte d'Ivoire

**Keywords:** Soil-transmitted helminths, Helminthiasis, Fecal calprotectin, Fecal occult blood, Intestinal morbidity

## Abstract

**Background:**

Infections with soil-transmitted helminths (STHs) may result in chronic inflammatory disorders affecting the human host. The objective of this study was to evaluate Fecal Calprotectin (FC) and Fecal Occult Blood (FOB) in individuals infected and non-infected with STHs to identify potential intestinal morbidity markers.

**Methods:**

Stool from participants diagnosed positive for *Trichuris trichiura* and concomitant STH infections from three countries was used to perform FC and FOB point-of-care assays. Simultaneously, identified STH negative participants underwent FC and FOB testing as controls. Potential associations between test results and determinants were analyzed using multivariable logistic regression.

**Findings:**

In total, 1034 *T. trichiura* infected cases (mostly light infections) and 157 STH negative controls were tested for FC and FOB. Among all participants tested, 18·5% had ≥ 50 µg/g FC concentration, while 14 (1·2%) were positive for FOB. No statistically significant association was found between *T. trichiura* infection or *Ascaris lumbricoides* co-infection and FC concentration, while an inverse association (odds ratio (OR): 0·45, 95% credible intervals (CrI): 0·26, 0·75) was found between hookworm co-infection and FC concentration. In Lao PDR, the proportion of participants in the ≥ 50 µg/g FC category was significantly higher in the oldest age category compared to the 5–11 years group (OR: 3·31, 95% CrI: 1·62, 7·24). Too few participants were found positive for FOB to derive any conclusions.

**Interpretation:**

Studies are needed to better understand the relationship between intestinal morbidity and STH infections. Suitable, standardized, low-cost markers of STH attributable morbidity to better monitor the impact of STH control interventions are necessary.

**Funding:**

BMGF (OPP1153928)

**Research in context**

**Evidence before this study**

Long-term infections with soil-transmitted helminths (STHs) contribute to substantial morbidity; however, evidence on appropriate point-of-care indicators of STH attributable morbidity is scarce. We searched in PubMed for all articles published before Nov 2, 2020 which mentioned “helminth”, *“Trichuris trichiura”*, “*Ascaris lumbricoides”*, or “hookworm” and “fecal calprotectin” or “fecal occult blood” in the abstract, without language restrictions. Contradictory findings result from a small body of evidence (nine studies) on the association between STH infection status, infection intensity and intestinal morbidity, using Fecal Calprotectin (FC) or Fecal Occult Blood (FOB) as indicators. Only one study used novel rapid diagnostic immunoassay tests for FOB detection, while none had a sufficient sample size to derive meaningful conclusions.

**Added value of this study**

This is the first large scale study testing FC and FOB as potential proxy markers for STH attributable intestinal morbidity in three different countries. No association between the presence of intestinal inflammation or mucosal bleeding, assessed with FC and FOB, and *T. trichiura* and *A. lumbricoides* infection status was found, while a negative association between FC concentration and hookworm infection was found.

**Implications of all the available evidence**

Further research should focus on the development and evaluation of potential morbidity markers, as the appropriate monitoring of STH attributable morbidity might become as important as diagnosing the infection itself. Thus, appropriate indicators of helminth attributable morbidity are still lacking.

Alt-text: Unlabelled box

## 1 Introduction

More than 1·5 billion people worldwide are infected with soil-transmitted helminths (STHs), namely *Ascaris lumbricoides*, hookworm (*Ancylostoma duodenale* and *Necator americanus*), and *Trichuris trichiura* [1]. Helminthiases typically affect the poorest and the most marginalized populations, particularly in tropical and subtropical regions, where access to water, sanitation, and hygiene is inadequate [2,3]. STH infections manifest, if left untreated, as generally asymptomatic chronic infections causing both concurrent and delayed-onset pathologies affecting the human host [4]. Chronic infections and/or infections of heavy intensities can result in malnutrition, malabsorption, reduced growth rate, intestinal obstruction, poor iron status, and iron deficiency anemia [4], [5], [6], [7], [8], [9], particularly in those on marginal diets [10]. Long-term consequences include subtle effects on cognition [11], educational performance [12], and school absenteeism [13], impacting individuals’ workforce potential, and economic progress among affected groups [14].

Embryonated *T. trichiura* eggs hatch in the small intestine and potentially attach to the mucosa in the large intestine, whereas larvae penetrate the epithelial cells for subsequent growing and molting into adult stages [15]. Adult worms embed their head part into a intracellular niche in the large intestine [16] causing petechial lesions, blotchy mucosal hemorrhage, and oozing leading to both mucosal and systemic immune responses [17], [18], [19]. Similarly, hookworm larvae use their cutting plates to attach to the mucosa to begin feeding and molting into adult worms, attributing to intestinal morbidity [20]. In contrast, *A. lumbricoides* feeds passively and never attaches directly to the mucosa [4,21].

The present goal of global control programs recommended by the World Health Organization (WHO), is to reduce morbidity with preventive chemotherapy (i.e. administration of albendazole or mebendazole without prior diagnosis) to at-risk populations (i.e. school-aged children and women of reproductive age) with accompanying improvements in access to clean water and sanitation to reduce worm burden associated morbidity [22]. Inherent with such control interventions is the necessity to define and validate indicators of helminth attributable morbidity. Even though in most cases morbidity is associated with infecton intensity [23], assessed by classic microscopic diagnosis detecting eggs in the feces, appropriate morbidity parameters are of pivotal importance as they provide additional information on the degree to which the STH infection affects the patient. This is particularly important after anthelmintic treatment, when intensity of infection has reduced markedly, but morbidity still is present [24]. A better clinical understanding of measurable reductions in STH attributable morbidity in response to anthelmintics is needed to appropriately shape and evaluate ongoing or future STH control programs [25].

Fecal biomarkers are promising non-invasive indicators possibly reflecting mucosal inflammation or damage, as molecules from the intestinal mucosa are transported in passing with the feces [26]. It is a well-known phenomenon that the occurrence of blood in feces can be indicative of pathologic changes, especially in the context of malignancies or inflammation [27].

Fecal Calprotectin (FC), a neutrophil cytoplasmic multimeric complex of the calcium-binding proteins, is abundant in neutrophil granulocytes, monocytes, and early stage macrophages [28,29]. Translocation of these cells into the intestinal mucosa and degranulation inside the intestinal lumen leads to increased secretion of FC as a response to local inflammation [30,31]. Given the fact that FC levels are stable in feces and not influenced by systemic infections, FC is an interesting biomarker for understanding the association between intestinal infection and inflammation by measuring the localized intestinal inflammation [19,[32], [33], [34]]. Therefore, FC is widely used across gastroenterology practices as a non-invasive surrogate marker for disease activity and response to treatment [35,36]. In the past years, its use in enteric infections is increasing, particularly as a correlative marker for clinical severity and in evaluating bacterial and viral pathogens [37].

Fecal blood is a late symptom of inflammatory tissue damage [30]. For example, blood loss due to *T. trichiura* infections has been estimated to be 5 µL per adult worm per day [38]. Thus, fecal occult blood (FOB) might be another candidate for a morbidity marker of helminthiasis. FOB tests have been previously used for identifying blood loss in hookworm infection, trichuriasis, and intestinal schistosomiasis [39], [40], [41], [42]. However, most studies used guaiac-based FOB tests that are known to be less sensitive than immunochemical assays [43].

Rapid immunological FC and FOB dipstick tests are non-invasive, easily preserved, and reliable diagnostic tools allowing immediate detection of FC for diagnosing intestinal inflammation and FOB for occult blood in the feces indicating intestinal morbidity [44]. Characteristics and applicability of field-appropriate diagnostic tools using proxy markers for STH morbidity are not well investigated to date [42]. Moreover, previous work on morbidity indicators is mostly limited to research on schistosomiasis causing gastrointestinal morbidity [[41], [42], [43],[45], [46], [47]]. Although, some case reports suggest mucosal damage as a consequence of STH infections [48,49], evidence on the association between infection and intestinal inflammation is lacking [8].

The objective of the current study was to investigate FC and FOB as potential STH gut morbidity markers as a tool to monitor the impact of community-level deworming. Using FC and FOB as biomarkers, we aimed at assessing the potential association between the presence of intestinal inflammation and STH infection status.

## 2 Methods

### 2.1 Study design

The presented data derive from a randomized controlled trial (RCT) assessing efficacies of ivermectin-albendazole and albendazole alone against *T. trichiura* and concomitant STH infections in participants aged 6–60 years. The study was conducted in communities in the Lagunes region in Côte d'Ivoire, in the Luang Prabang Province in Lao PDR, and in South Pemba on Pemba Island, Tanzania during the screening period (Nov 2018 to Dec 2019) of the trial. Prior to study initiation, ethical clearance was granted by the Ethics Committee of Northwestern and Central Switzerland (EKNZ; reference no: BASEC Nr Req-2018–00494), the Zanzibar Medical Research and Ethics Committee (ZAMREC, reference no.: ZAMREC/0003/Feb/2018), the Comité National d’Éthique et de la Recherche, Ministère de la Santé et de Lutte contre le SIDA (reference no.: 088–18/MSHP/CNESVS-km), the Direction de la Pharmacie, du Médicament et des Laboratoires (reference no. ECCI00918) in Côte d'Ivoire, and the National Ethics Committee for Health Research, Ministry of Health in Lao PDR (reference no. 093/NECHR). Trial and study details are summarized in the published trial protocol [50] and in the trial registration (clinicaltrials.gov, reference: NCT03527732, date assigned: 17 May 2018).

Prior to study enrollment, all inhabitants of the chosen villages were invited to information sessions at local places, during which the research staff explained the purpose and procedures of this study, as well as the potential benefits and risks of participation. Written informed consent was obtained from adults and parents or legal guardians of children below the age of adulthood (21 years in Côte d'Ivoire and 18 years in Lao PDR and Pemba Island). Children aged below the age of adulthood gave written assent (Côte d'Ivoire) or oral assent (Lao PDR and Pemba Island).

### 2.2 Study procedures

A short census was conducted at the start of screening, during which the name, sex, age, and village name were recorded for all participants. Consenting and eligible participants (aged 6–60, as the only inclusion criteria for screening) were asked to provide two fresh morning stool samples (as per inclusion criteria), preferably on consecutive days, in containers labelled with their assigned unique ID. Collected stool samples were kept in a cool box containing ice packs while being transported to the field laboratory.

Duplicate Kato-Katz thick smears (2 × 41·7 mg of stool) were prepared and examined under a microscope by experienced laboratory technicians for species-specific diagnosis of STH ova (i.e. *T. trichiura, A. lumbricoides,* and hookworm) within 60 min after preparation to avoid over-clearing of hookworm eggs [51] following the World Health Organization (WHO) standard procedures [52]. Additionally, in Lao PDR, Kato-Katz thick smear slides were examined for *Opisthorchis viverrini* infection and infections of *Strongyloides stercoralis* were classified as larvae-positive or negative using the Baermann technique [53]. To assure high quality of the microscopic evaluation, 10% of all Kato-Katz slides were randomly chosen, re-labeled, and re-examined for *A. lumbricoides* and *T. trichiura*. In brief, microscopic results were considered inconsistent if there was a difference in presence/absence of a specific helminth species, or if differences in egg counts exceeded (i) 10 eggs for Kato-Katz thick smears with ≤ 100 eggs, or (ii) exceeded 20% for Kato-Katz thick smears with > 100 eggs. In case of discrepancies between the original and the quality control read, slides were read by a third independent microscopist and results were discussed until consensus was reached [54].

Of those found positive for *T. trichiura*, a random subsample of unique ID numbers (generated by a co-investigator not involved in the laboratory work) were chosen to undergo FC and FOB testing the same day. At the same time, of those participants found negative for *T. trichiura, A. lumbricoides*, and hookworm, a subsample of approximately 50 participants were chosen at random (using a list generated in the same manner as described above) in each setting to undergo FC and FOB testing. Participants who were found not to be infected with *T. trichiura*, but were infected with *A. lumbricoides* and/or hookworm infections were not included in this study. All included participants donated two fecal samples in order to increase the precision of the STH infection result. On Pemba Island, the first of the two fecal sample was used for subsequent rapid diagnostic testing (RDT). Due to the low prevalence of *T. trichiura* infections in the communities chosen in Lao PDR and Côte d'Ivoire, it was decided to use the second donated stool sample to ensure participants in this study would also be included in the larger trial, which had a minimum infection intensity cut-off of 100 *T. trichiura* eggs per gram of stool.

Cases that were subsequently enrolled in the clinical trial, were clinically examined by a physician and their height, weight, and hemoglobin levels recorded. However, negative controls, whom were not eligible for trial inclusion, did not undergo clinical examination.

### 2.3 Point-of-care tests: fecal calprotectin and fecal occult blood

A semi-quantitative chromatographic immunoassay (Actim® Fecal Calprotectin test, Medix Biochemica, Finland) was applied for FC detection according to the manufacturer's instructions. In brief, the participant number was first written on the Specimen Dilution Buffer tube (3 mL) before unscrewing. The sampling stick, attached to the cap, was first twisted into different places of the fecal sample in order to make sure that both slits at the head of the stick contained stool. The sampling stick was put back in the tube and shaken to suspend the feces in the buffer. A dipstick was then inserted into the perforation area of the buffer tube before inverting the tube by 90° for two seconds. The tube was then placed on a flat surface in an upright position. Results were read after ten minutes with one test line indicating a valid test result with a FC level < 50 µg/g, two lines indicating a level of 50–200 µg/g, and three lines indicating a level > 200 µg/g of FC concentration in the test specimen based on reference values provided by the manufacturer. FC levels < 50 µg/g were interpreted as no inflammation, 50–200 µg/g of FC as possible inflammation and > 200 µg/g of FC as likely active inflammation, similar to suggestions by Bressler et al [55].

A simple immunochemical test was applied for FOB (Actim® Fecal Blood test, Medix Biochemica, Finland) detection, following the manufacturer's instructions. Unlike traditional guaiac tests, this test is based on highly specific monoclonal antibodies that only detect human hemoglobin, thus, food substances containing hemoglobin or peroxidase activity do not influence test results. Test procedure was similar to the aforementioned FC test, however; positive results were readable immediately as soon as two blue lines appeared, negative samples remained with only one blue line after ten minutes. Detailed test characteristics (e.g. sensitivity and specificity) are summarized by the manufacturer and published online [56].

Fecal rapid tests were conducted by laboratory personnel not involved in assessing parasitological data and masked to the identity of participants providing samples. The results from each test were then recorded on a personal log form by the technician involved in the fecal rapid diagnostic testing. If no control line appeared, the result was recorded as invalid and was repeated with a new dipstick.

### 2.4 Outcomes

The objective of this study was to evaluate FC and FOB as potential STH gut morbidity markers. Adjusted odds ratios of FC and FOB values were calculated for STH infected participants compared to STH negative controls.

### 2.5 Sample size

We aimed for a total sample size of 1050: 300 *T. trichiura* positive individuals and 50 individuals negative for all STH infections per country. This study was conducted within the framework of a RCT; therefore, no separate sample size calculation has been conducted. Since prevalence of FC and FOB is unlikely very rare and varies drastically by setting, a sample size of above 1000 was deemed to be sufficient. Moreover, experience has shown that the study would be sufficiently powered unless the outcome or the exposure is rare. We estimated we would be able to detect a halving of risk with at least 80% power if the prevalence in the non-exposed group is at least 10% or higher.

### 2.6 Statistical analysis

Data were double entered into EpiInfo 3.5.4 by two independent data clerks and crosschecked using the data compare tool of EpiInfo 3.5.4 Any discrepancies between the two datasets were resolved by consulting the original hardcopy. Descriptive analysis was done in Stata IC 15 (StataCorp.; College Station, TX), whereas all statistical estimations were performed in R 3.5.1 (RStudio, PBC, Boston, MA). Proportions of infection with *T. trichiura* and co-infections with *A. lumbricoides* and/or hookworm were assessed for all participants in all three settings, while for Lao PDR *O. viverrini* and *S. stercoralis* co-infections were additionally assessed.

Results from the duplicate Kato-Katz smears from each of the two stool samples were summed and multiplied by a factor of six to be expressed as mean egg count per gram (EPG) of feces. Associations between FC with participant's age, sex, country, and parasitic infections was assessed using Bayesian logistic regression with logit link to estimate odds ratios (ORs) with corresponding 95% credible intervals (CrI). The Bayesian framework - as implemented in the R package 'rstanarm' (v 2.19.3) - was preferred to avoid potential quasi separation problems. We used the default weakly informative prior distributions for all parameters, i.e. normal priors with mean 0 and standard deviation 2·5. The Markov chain Monte Carlo alogorithm drew 1000 samples from 4 chains after a warm-up of additional 1000 samples per chain. Convergence was assessed by the R-hat statistic. Smoothing lines in figures were predicted via generalized additive models for binomial data. Due to the low number of individuals (*n* = 14/1189) testing positive for FOB, it was decided to forego formal statistical testing and present the results descriptively.

### 2.7 Role of the funding source

The funder of the study had no role in study design, data collection, data analysis, data interpretation, or writing of the report. The corresponding author had full access to all the data in the study and had final responsibility for the decision to submit for publication.

## 3 Results

### 3.1 Participants characteristics

**Fig. 1:**
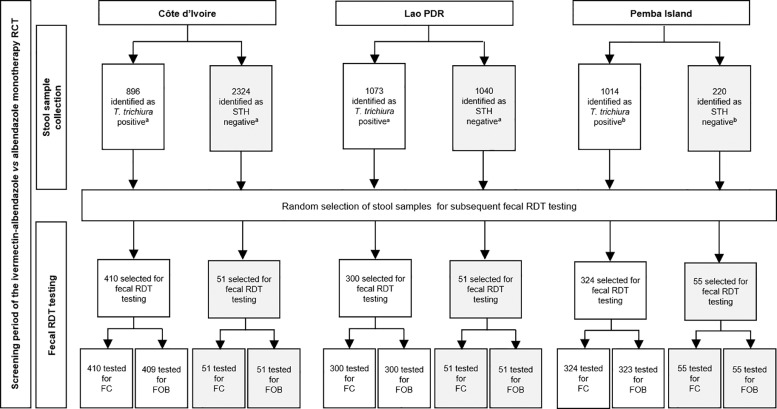
Study design. Abbreviations: *T. trichiura, Trichuris* t*richiura*; FC, Fecal Calprotectin, FOB; Fecal Occult Blood; Lao PDR, Lao People's Democratic Republic; RCT, Randomized Controlled Trial; RDT, Rapid Diagnostic Test; STH, Soil-transmitted helminth ^a^ Positive or negative according to the first and second stool sample; second stool sample was used for subsequent fecal RDT ^b^ Positive or negative according to the first stool sample, which was used for subsequent fecal RDT on the same day.

Fig. 1 shows the study design by setting. Out of the 6567 participants screened, 2983 (45·4%) were positive for *T. trichiura* infection and 3584 (54·6%) were negative for all STH infections. From these, 1034 of the 2983 *T. trichiura* positive cases and 157 of the STH negative cases were randomly selected for FC and FOB testing.

Baseline parasitological and demographic characteristics of participants surveyed for FC and FOB are summarized in Table 1. Of those participants infected with *T. trichiura* (*n* = 1034), 51·5% were female, while the mean age of those was 18·7 (±15·0) years. The identified STH negative participants (*n* = 157) were slightly older with a mean age of 30·1 (± 16·2), while 66·2% of those participants were female.

**Table 1:** Baseline characteristics of *T. trichiura* positive cases and STH negative controls.

	*T. trichiura* positive, n (%)	STH negative, n (%)
Total of participants included, N. (%)^a^	1034 (86·8)	157 (13·2)
Females, n (%)	533 (51·5)	104 (66·2)
Mean age, years (SD)	18·7 (15·0)	30·1 (16·2)
Mean BMI, kg/m^2^ (SD)^b^	18·1 (4·6)	NA
Mean hemoglobin, g/L (SD)^b^	124·6 (14·5)	NA
*T.trichiura* infection		NA
Geometric mean EPG	492·0	
Infection intensity^c^, n (%)		
Light	784 (75·8)	
Moderate	240 (23·2)	
Heavy	10 (1·0)	
*A. lumbricoides* co-infection		NA
Infected, n (%)	345 (33·3)	
Geometric mean EPG	4317·9	
Infection intensity^c^, n (%)		
Light	167 (48·4)	
Moderate	150 (43·5)	
Heavy	28 (8·1)	
Hookworm co-infection		NA
Infected, n (%)	338 (32·7)	
Geometric mean EPG	559·4	
vInfection intensity^c^, n (%)		
Light	253 (74·9)	
Moderate	49 (14·5)	
Heavy	36 (10·7)	
*S. stercoralis* co-infection^d^		NA
Infected/Surveyed, n (%)	49/277 (17·7)	
*O. viverrini* co-infection^d^		NA
Infected, n (%)	54/300 (18·0)	

Abbreviations: *A. lumbricoides, Ascaris lumbricoides*; BMI, body mass index; EPG, eggs per gram; NA, not applicable; *O. viverrini, Opisthorchis viverrini;* SD, standard deviation; *S. stercoralis, Strongyloides stercoralis; T. trichiura, Trichuris trichiura*.

a Baseline characteristics were assessed for 1191 participants surveyed for FC. Additionally, 2 participants missed FOB testing, however, baseline characteristics between FC and FOB are thus very similar.

b BMI and hemoglobin was only assessed in participants attending subsequent clinical examination.

c Infection intensities were classified according to WHO recommendations, based on guidelines established by Montresor et al. (1998).

d *O. viverrini* and *S. stercoralis* co-infections were only assessed in Lao PDR.

In addition to *T. trichiura* infection, 345 (33·3%) participants were co-infected with *A. lumbricoides*, while 338 (32·7%) participants harbored a hookworm co-infection. Among those participants in Lao PDR, 18·0% were found to be co-infected with *O. viverrini* and 17·7% with *S. stercoralis*. Mean body mass index (BMI) and mean hemoglobin concentration were 18·1 (±4·6) kg/m² and 124·6 (±14·5) g/L, respectively.

Median EPGs were 402, 5508, and 759 for *T. trichiura* infections and *A. lumbricoides* and hookworm co-infections, respectively (Table 2). Table 3 shows that the EPG distribution differs among settings with Pemba Island, having a higher median EPG (558, IQR 330–1224) than Côte d'Ivoire (median EPG: 408, IQR 198–1068), and Lao PDR (median EPG: 252, IQR 150–543) for those with *T. trichiura* infection. *A. lumbricoides* co-infection was found in 35·9%, 36·0%, and 27·8% of study participants in Côte d'Ivoire, Lao PDR, and Pemba Island, respectively; though the proportion of *T. trichiura* infected participants with hookworm co-infection was higher in Lao PDR (91·7%) compared to Côte d'Ivoire (7·6%) and Pemba Island (9·9%).

**Table 2:** Test results of fecal calprotectin and fecal occult blood in *T. trichiura* positive and STH negative participants.

			Fecal calprotectin			Fecal occult blood	Total N(%)
		< 50 µg/g	50–200 µg/g	> 200 µg/g	Negative	Positive	
Number of test results, n (%)		971 (81·5)	167 (14·0)	53 (4·5)	1175 (98·8)	14 (1·2)	1191^a^ (100)
***T. trichiura*****positive, n (%)**		842 (86·7)	149 (12·5)	43 (3·6)	1020 (98·8)	12 (1·2)	1034 (100)
Females, n (%)		436 (81·8)	77 (14·4)	20 (3·8)	520 (97·9)	11 (2·1)	533 (100)
Mean age, years (SD)		18·0 (14·3)	22·1 (17·5)	21·1 (17·4)	18·6 (14·9)	29·6 (18·2)	18·8 (15·0)
Mean BMI, kg/m^2^ (SD)^b^		18·0 (4·5)	18·9 (5·1)	18·0 (4·5)	18·1 (4·6)	19·2 (3·7)	18·1 (4·6)
Mean hemoglobin, g/L (SD)^c^		124·6 (14·5)	124·6 (14·3)	126·0 (14·9)	124·6 (14·4)	123·3 (20·6)	124·6 (14·5)
Median EPG (IQR)		408	360	390	405	279	402
		(216–948)	(210–966)	(186–720)	(210–960)	(138–432)	(210–948)
**Co-infections with*****A. lumbricoides***							
Positive (%)		273 (78·9)	54 (15·6)	19 (5·5)	343 (99·1)	2 (0·9)	346 (100)
Median EPG (IQR)		4716	9864	10,716	5508	12,861	5508
		(1212–14,376)	(1830–32,802)	(4014–25,362)	(1332–18,462)	(360–25,362)	(1332–18,462)
**Co-infections with hookworm**							
Positive (%)		271 (79·9)	49 (14·5)	19 (5·6)	330 (97·3)	9 (2·7)	339 (100)
Median EPG (IQR)		714	954	522	729	1158	759
		(186–1974)	(492–2064)	(210–2556)	(198–1968)	(252–6042)	(204–2064)
**Co-infections of*****T. trichiura***							
with 1 other STH		442 (79·0)	87 (15·5)	31 (5·5)	548 (98·2)	10 (1·8)	560 (100)
with 2 other STHs		101 (82·1)	15 (12·2)	7 (5·7)	122 (12·0)	1 (8·3)	123 (100)
with *O. viverrini*^d^		43 (79·6)	8 (14·8)	3 (5·6)	53 (98·1)	1 (1·9)	54 (100)
with *S. stercoralis*^d^		39 (79·6)	7 (14·3)	3 (6·1)	48 (98·0)	1 (2·0)	49 (100)
						
**STH negative, n (%)**		**129 (82·2)**	**18 (11·4)**	**10 (6·4)**	**155 (98·7)**	**2 (1·3)**	**157 (100)**
Females (%)		89 (69·0)	9 (50·0)	6 (60·0)	104 (67·1)	0 (0·0)	104 (100)
Mean age, years (SD)		31·3 (15·4)	32·4 (20·5)	20·9 (16·1)	30·1 (16·1)	30·5 (29·0)	21·1 (17·4)

Abbreviations: *A. lumbricoides, Ascaris lumbricoides*; BMI, body mass index; EPG, eggs per gram; IQR, interquartile range; *O. viverrini, Opisthorchis viverrini;* SD, standard deviation; *S. stercoralis, Strongyloides stercoralis; T. trichiura, Trichuris trichiura*.

a 1191 FC test results and 1189 FOB test results were obtained, as not enough stool was collected from two individuals to conduct FOB tests.

b Data was only collected in 954 individuals for FC and 953 individuals for FOB, as STH negative and other possibly eligible participants did not attend subsequent clinical examination.

c Data was only collected in 957 individuals for FC and 956 individuals for FOB, as STH negative and other possibly eligible participants did not attend subsequent clinical examination.

d *O. viverrini* and *S. stercoralis* infections were only assessed in Lao PDR.

**Table 3:** Age, sex, and co-infection for participants surveyed for fecal calprotectin and fecal occult blood by country.

	Côte d'Ivoire		Lao PDR		Pemba Island	
	*T. trichiura* positive	all STH negative	*T. trichiura* positive	all STH negative	*T. trichiura* positive	all STH negative
Participants, n	410	51^a^	300	51^b^	324	55
Mean age in years (SD)	16·3 (13·6)	31·5 (14·8)	28·3 (17·5)	24·6 (17·5)	13·0 (8·8)	24·9 (14·3)
Age categories						
5–11 years (%)	251 (61·2)	8 (16·0)	92 (30·7)	20 (40·0)	182 (56·2)	2 (3·6)
12–34 years (%)	99 (24·2)	18 (36·0)	91 (30·3)	11 (22·0)	126 (38·9)	22 (40·0)
35–64 years (%)	60 (14·6)	24 (48·0)	117 (39·0)	19 (38·0)	16 (4·9)	31 (56·4)
Females (%)	209 (51·0)	29 (58·0)	157 (52·3)	31 (62·0)	167 (51·5)	43 (78·2)
						
Median EPG (IQR))	408 (198–1068)	0 (0)	252 (150–543)	0 (0)	558 (330–1224)	0 (0)
						
*A. lumbricoides* infection (%)	147 (35·9)	0 (0)	108 (36·0)	1 (2·0)^b^	90 (27·8)	0 (0)
Hookworm infection (%)	31 (7·6)	1 (2·0)^a^	275 (91·7)	0 (0)	32 (9·9)	0 (0)
*O. viverrini* infection (%)	ND	ND	54 (18·0)	1 (2·0)	ND	ND
*S. stercoralis* infection (%)	ND	ND	49 (17·7)	0 (0·0)	ND	ND
						
Fecal calprotectin						
< 50 µg/g (%)	311 (75·9)	40 (80·0)	231 (77·0)	33 (66·0)	300 (92·6)	55 (100·0)
≥ 50 µg/g (%)	99 (24·1)	10 (20·0)	69 (23·0)	17 (34·0)	24 (7·4)	0 (0)
						
Fecal occult blood^c^						
Positive (%)	2 (0·5)	0 (0)	9 (3·0)	2 (4·0)	1 (0·3)	0 (0)
Negative (%)	407 (99·5)	50 (100·0)	291 (97·0)	48 (96·0)	322 (99·7)	55 (100·0)

Abbreviations: *A. lumbricoides, Ascaris lumbricoides*; ND, not determined; *O. viverrini, Opisthorchis viverrini*; SD, standard deviation; STH, soil-transmitted helminths; *S. stercoralis, Strongyloides stercoralis; T. trichiura, Trichuris trichiura*.

a 1 of 51 participants was wrongly assigned to a STH negative sample. The hookworm co-infection was of light intensity.

b 1 of 51 participants was wrongly assigned to a STH negative sample. The *A. lumbricoides* co-infection was of light intensity.

c Only 409 and 323 assessed in Côte d'Ivoire and Pemba Island, respectively.

### 3.2 Fecal rapid diagnostic test results

As shown in Table 2, of those diagnosed with *T. trichiura* (*n* = 1034), most participants (842 (86·7%)) were found to have a normal FC concentration of < 50 µg/g, while 149 (12·5%) were found to have elevated FC concentrations of 50–200 µg/g. Only a few participants (43 (3·6%)) were identified with high (> 200 µg/g) FC levels. Most participants harboring a co-infection with *A. lumbricoides* (78·9%) or hookworm (79·0%) were found to have a normal FC concentration of < 50 µg/g.

Negative controls showed a similar distribution of FC levels; the majority 129 (82·2%) had a normal FC concentration of < 50 µg/g, while 18 (11·4%) were identified with FC concentrations of 50–200 µg/g and 10 (6·4%) with high FC concentrations of > 200 µg/g. Age, Sex, BMI, and hemoglobin level were similar among the different FC levels.

During the study, 1189 participants were surveyed for FOB, while only a minority of 14 (1·2%) participants were found to be positive. Most of the participants (78·6%) tested positive for FOB were found in the Asian setting. Country specific study results are summarized in Table 3.

**Fig. 2:**
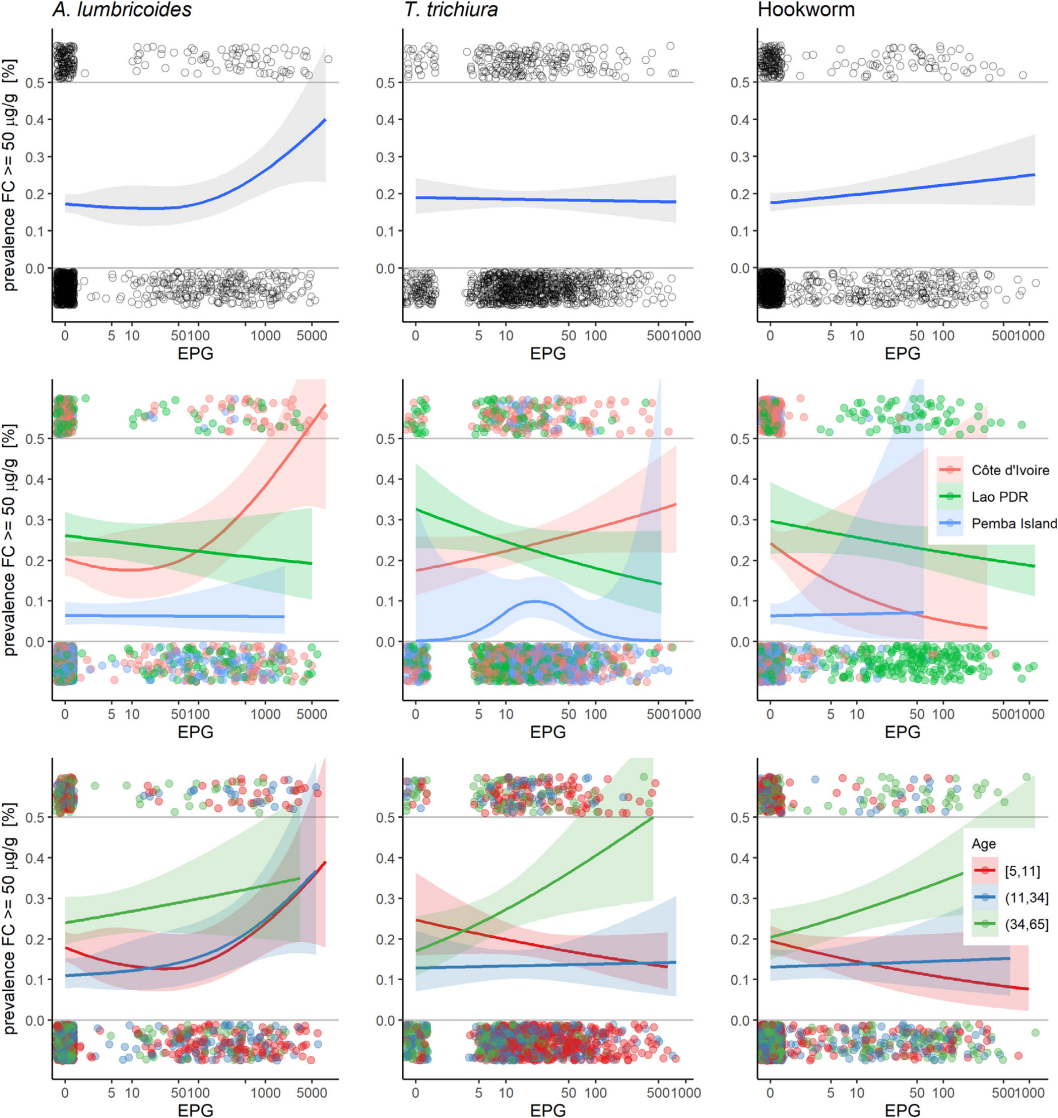
Prevalence of ≥ 50 µg/g FC concentration by EPG for *Trichiura trichuris* infections*, Ascaris lumbricoides,* and hookworm co-infections, and, in addition, by country (middle row), and age group (bottom row). Abbreviations: *A. lumbricoides, Ascaris lumbricoides;* EPG, eggs per gram; FC, fecal calprotectin; *T. trichiura, Trichuris trichiura*.

Fig. 2 shows the proportion of ≥ 50 µg/g concentration of FC by EPG for each STH and, additionally, by country and age group. For *T. trichiura* infections and hookworm co-infections, FC proportion of ≥ 50 µg/g remains flat as EPG increases; while there is an increase in percentage of ≥ 50 µg/g starting at 100 EPG for *A. lumbricoides* co-infections.

Results of multivariable logistic regression models are summarized in Table 4, while country-specific results are shown in S1 Table (Supplementary Table 1). When results from all three countries were combined, no association was found between FC and infection with *T. trichiura* and co-infection with *A. lumbricoides*, while hookworm co-infection was associated with lower odds (OR: 0·45, 95% CrI: 0·26, 0·75). Country was the greatest overall predictor of FC concentration with Lao PDR having a higher odds (OR: 1·77, 95% CrI: 1·09, 2·97)) of ≥ 50 µg/g FC concentration and Pemba Island having a lower odds (OR: 0·23, 95% CrI: 0·14, 0·37) of ≥ 50 µg/g FC concentration when compared, respectively, to Côte d'Ivoire. Older participants (ages 36–64 years) had a statistically significant higher odds of ≥ 50 µg/g FC concentration when compared to participants ages 5–11 years (OR: 1·49, 95% CrI: 1·00, 2·20)). In Lao PDR, the higher odds of ≥ 50 µg/g FC concentration amongst older participants was also seen (OR: 3·31, 95% CrI: 1·62, 7·24 for 36–64 age group). The raw data suggests a country × *T. trichiura* interaction. Therefore, an additional model including the interaction terms was fitted, which can be found in S2 Table (Supplementary Table 2). However, the estimated odds ratios for age categories, sex, and co-infections changed only slightly.

**Table 4:** Determinants for fecal calprotectin levels in fecal stool samples. Presented are odds ratios and 95% credible intervals estimated by multivariable logistic regression.^a^

Variable	FC ≥ 50 µg/g	FC < 50 µg/g	OR	95% CrI
All participants	220/1191 (18·5%)	971/1191 (81·5%)		
**Country**				
Côte d'Ivoire	109 (23·6%)	352 (76·4%)	ref	ref
Lao PDR	86 (24·6%)	264 (75·4%)	1·77	1·09, 2·97
Pemba Island	24 (6·3%)	355 (93·7%)	0·23	0·14, 0·37
**Age categories**				
5–11 years	102 (18·4%)	453 (81·6%)	ref	ref
12–34 years	49 (13·8%)	305 (86·2%)	0·78	0·52, 1·16
35–64 years	69 (24·5%)	213 (75·5%)	1·49	1·00, 2·20
**Sex**				
Female	112 (17·6%)	525 (82·4%)	ref	ref
Male	108 (19·5%)	446 (80·5%)	1·12	0·82, 1·50
***T. trichiura*****infection**				
Negative	28 (17·8%)	129 (82·2%)	ref	ref
Positive	192 (18·6%)	842 (81·4%)	1·48	0·87, 2·60
***A. lumbricoides*****co-infection**				
Negative	147 (17·4%)	698 (82·6%)	ref	ref
Positive	73 (21·1%)	273 (78·9%)	1·19	0·85, 1·66
**Hookworm co-infection**				
Negative	152 (35·9%)	271 (64·1%)	ref	ref
Positive	68 (8·9%)	700 (91·1%)	0·45	0·26, 0·75

Abbreviations: *A. lumbricoides, Ascaris lumbricoides*; CrI, credible intervals; FC, fecal calprotectin; OR, odds ratio; ref, reference group; *T. trichiura, Trichuris trichiura*.

a Regression model includes country, age, sex, *T. trichiura, A. lumbricoides,* and hookworm infection status.

## 4 Discussion

We applied FC and FOB tests to assess the relationship between STH infections and intestinal inflammation or mucosal bleeding in three different countries. It is of pivotal importance to find an affordable, standardized and simple point-of-care test to assess STH attributable morbidity to better survey control interventions. Evidence on the association between local inflammation and STH infections is scarce; however, key problems with much of the literature are the generally small sample sizes [40,57,58] and the use of different biomarkers [19] for intestinal inflammation leading to contradictory results.

We found no associations of FC and FOB with *T. trichiura* infection and *A. lumbricoides* co*-*infection status and intensity in each of the three settings tested, demonstrating that FC and FOB are not good proxy markers for STH attributable gut morbidity. Known immunoregulatory properties of STHs have shown a down-regulation of host response to limit inflammation and tissue damage, which may explain our findings [59,60]. Albeit our results show a slight protective effect of hookworm co-infection, this might be attributed to the hypothesis that hookworm infection causes a dampening effect on FC levels by inhibiting neutrophils, the main calprotectin-producing cell type [61]. Of note, *N. americanus* is expected to be the predominant hookworm species in our settings, ingesting around 0·001 mL blood per day [62], [63], [64], [65] Our results suggest age and setting are greater predictors of FC concentration than the presence of helminth infection. Though age has been thoroughly established as a predictor for inflammation in the body [66,67], the root cause of why setting is predictive of high calprotectin is yet to be determined although different diets and exercise might play a role [68].

Our findings add to the small body of evidence currently available on FC concentration and helminth infections. The results we found substantiate on a larger scale the previous findings of de Gier and colleagues, who reported no association between FC concentration and STH infection in 2018 [19]. Additionally, de Gier et al. reported no association between hookworm and FC concentration at baseline or at seven months follow-up [69]. Cepon-Robins et al. similarly observed in a small sample size among the Shuar of Amazonian Ecuador that the relationship between infection and intestinal inflammation were age- and species-specific. These researchers found children singly infected with *T. trichiura* to have significantly lower FC levels, regardless their infection intensity, while no significant relationships were found among adults [57].

Surprisingly, we only found 12 FOB positive results among all STH infected participants. Though we did not formally apply a statistical test for FOB, due to the low number of positive test results, our findings correlate fairly well with Raj et al. as they also did not find a significant difference in the rate of FOB between *T. trichiura* or *A. lumbricoides* positive and negative children [70]. These findings are supported by Wakid who did not detect significant evidence on intestinal parasitic infections, including STHs, and positive FOB tests [71]. In contrast, the findings of Kanzaria et al. and Wanachiwanawin et al. support a relationship between FOB and moderate and heavy *T. trichiura* infections only [40,43].

Different findings were documented for schistosomiasis, a strong association between prevalence and intensity of *Schistosoma mansoni* infection and FOB was observed after repeated treatment over a period of one year in a cohort of young children [41]. These findings seem to be supported by Bustinduy et al. who found a significant correlation between FOB and moderate and heavy egg intensities of *S. mansoni* infection [46]. Moreover, Kanzaria et al. found a positive correlation between FOB and *Schistosoma japonicum* [43]. However, comparison to these studies on schistosomiasis needs to be interpreted with caution, as the host-parasite interaction differs compared to STHs. In the case of intestinal schistosomiasis, the disease is progressed by the chronic and downregulated granulomatous response to entrapped eggs causing polypsosis and pseudopolypsosis leading to rectal bleeding [72].

According to our findings, we suggest that intestinal inflammation and mucosal bleeding caused by STH infections potentially are very low-grade. There might be an age attributable reduction in the rate of parasite establishment, survival, and fecundity due to acquired immunity [73]. Moreover, immune system response to presence of STH ova might differ between the chosen settings. Additionally, blood loss in STH infected individuals might particularly result from the active feeding by adult worms, rather than the leakage around the parasite's attachment site in the gut, leading to decreased levels of hemoglobin instead of blood loss in feces [74]. Therefore, it remains challenging measuring morbidity in the context of STH infection and treatment, which is in agreement with Bogoch et al., who conducted a detailed clinical examination to determine whether STH cause measurable morbidity [75], which was not the case.

It is plausible that a number of limitations might have influenced the results obtained. Some controversy remains on the optimal cut-off values. Historical data on FC levels derive from delevoped countries and evidence on normal FC levels in populations equivalent to our study population is still limited, hampering interpretation of our data. Little evidence suggests to consider FC levels of 50–200 µg/g as normal in people of African-Caribbean descent; however, repeated tests at different time points in several settings would need to be done to strengthen this finding [76]. Similar to most point-of-care RDTs, precision can be limited to ease interpretation and dissemination of results. A meta-analysis by Lin et al. found that in regular FC RDTs, sensitivity is decreasing with higher FC levels, while specificity is increasing [77]. Moreover, the limit of detection of the FC test used in this study was > 200 µg/g, however, especially values > 500 µg/g seem to highly predict pathological findings [76]. As most STH infections of our study population were of light infection intensities, we might have missed an association between heavy infections and intestinal inflammation (FC) or mucosal bleeding (FOB). Furthermore, our study design was restricted to cases of *T. trichiura,* which limits the interpretation for *A. lumbricoides* and hookworm infections to co-infections rather than single infections, which may affect the interpretation of the results. As FC and FOB tests are not disease-specific, we were not able to control for chronic gastrointestinal diseases, malaria, schistosomiasis, and other confounding infective agents, such as bacterial pathogens. In addition, due to monetary constraints, we only analysed one fecal sample for FC/FOB per participant and a sufficient internal quality control method for FC/FOB testing could not be established. Future studies should establish such controls, whenever possible, by partially re-labeling and re-examining results of tests. Moreover, as in many multicountry research, various challenges arose across multiple settings. Differences in diet, culture, and social norms were unable to be accounted for and could potentially bias results. In addition, varying seasonality, host susceptibility, and parasite strains would have an uncontrollable impact on the findings of this study. In spite of these challenges, the results of this study add to the expanding body of literature of STH attributable morbidity. Lastly, the generalizability of our findings are limited to populations in resource-limited settings with recurring and chronic STH infections.

In conclusion, we were not able to detect intestinal morbidity in our study population using FC and FOB as proxy markers. Other potential markers of intestinal inflammation including antitrypsin, eosinophilic protein X, TNFα, or lysozyme have been suggested for diagnosing inflammatory bowel disease, which may be expanded to test for STH attributable intestinal morbidity. However, these test suffer from low diagnostic performance or have not been investigated well enough [78]. Furthermore, markers assessing other facets of STH attributable intestinal morbidity, such as mucosal changes, are needed. Thus, field applicable morbidity markers for STH infections need to be further explored to appropriately monitor and evaluate current global control strategies as monitoring levels of morbidity might become as important as diagnosing the infection itself.

## 5 Author contributions

EH and JK planned and designed the study; CP, LK, SW, SS, SMA, ShMA, JTC, EH, and JK conducted the study; JH and CP did the formal analyses; LK, CP, and JK interpreted the study data and drafted the manuscript; SW and EH assisted with revising and editing. All authors read and approved the final version of the manuscript and took responsibility to submit this manuscript for publication.

## Appendix Supplementary materials

Image, application 1
