# Supplementary material for: Assessment of fecal calprotectin and fecal occult blood as point-of-care markers for soil-transmitted helminth attributable intestinal morbidity in a case-control substudy conducted in Côte d'Ivoire, Lao PDR and Pemba Island, Tanzania
Source: eClinicalMedicine. 2021 Jan 30;32:100724. doi: 10.1016/j.eclinm.2021.100724 (PMC7851339; doi:10.1016/j.eclinm.2021.100724)
Supplement: Supplementary file 1 [file mmc1.docx]

Supplementary Material

S1 Table. Determinants for fecal calprotectin levels in fecal stool samples for Côte d’Ivoire, Lao PDR and Pemba Island. Presented are odds ratios and 95% credible intervals estimated by multivariable logistic regression.^a^

| **Variable** | **FC ≥ 50 µg/mg** | **FC < 50 µg/mg** | **OR** | **95% CrI** | **Sensitivity ^b^** |
| --- | --- | --- | --- | --- | --- |
| **Côte d'Ivoire** | n: 109 | n: 352 |  |  |  |
| **Age** |  |  |  |  |  |
| 5-11 years | 66 (25·5%) | 193 (74·5%) | ref | ref |  |
| 12-34 years | 22 (18·8%) | 95 (81·2%) | 0·67 | 0·36, 1·19 |  |
| 35-64 years | 21 (24·7%) | 64 (75·3%) | 1·02 | 0·54, 1·85 |  |
| **Sex** |  |  |  |  |  |
| Female | 52 (21·8%) | 187 (78·2%) | ref | ref |  |
| Male | 57 (25·7%) | 165 (74·3%) | 1·20 | 0·77, 1·82 |  |
| ***Trichuris trichiura* infection** |  |  |  |  |  |
| Negative | 10 (19·6%) | 41 (80·4%) | ref | ref |  |
| Positive | 99 (24·1%) | 311 (75·9%) | 1·13 | 0·51, 2·60 | 90·8% |
| ***Ascaris lumbricoides* infection** |  |  |  |  |  |
| Negative | 66 (21·0%) | 248 (79·0%) | ref | ref |  |
| Positive | 43 (29·3%) | 104 (70·7%) | 1·52 | 0·95, 2·39 | 39·4% |
| **Hookworm infection** |  |  |  |  |  |
| Negative | 105 (24·5%) | 324 (75·5%) | ref | ref |  |
| Positive | 4 (12·5%) | 28 (87·5%) | 0·45 | 0·13, 1·19 | 3·6% |
| **Lao PDR** | n: 87 | n: 264 |  |  |  |
| **Age** |  |  |  |  |  |
| 5-11 years | 22 (19·6%) | 90 (80·4%) | ref | ref |  |
| 12-34 years | 18 (17·6%) | 84 (82·4%) | 1·07 | 0·47, 2·44 |  |
| 35-64 years | 47 (34·3%) | 90 (65·7%) | 3·31 | 1·62, 7·24 |  |
| **Sex** |  |  |  |  |  |
| Female | 48 (25·5%) | 140 (74·5%) | ref | ref |  |
| Male | 39 (23·9%) | 124 (76·1%) | 0·97 | 0·53, 1·76 |  |
| ***Trichuris trichiura* infection** |  |  |  |  |  |
| Negative | 18 (35·3%) | 33 (64·7%) | ref | ref |  |
| Positive | 69 (23·0%) | 231 (77·0%) | 0·22 | 0·00, 7·09 | 79·3% |
| ***Ascaris lumbricoides* infection** |  |  |  |  |  |
| Negative | 63 (26·0%) | 179 (74·0%) | ref | ref |  |
| Positive | 24 (22·0%) | 85 (78·0%) | 0·88 | 0·49, 1·60 | 27·6% |
| **Hookworm infection** |  |  |  |  |  |
| Negative | 25 (32·9%) | 51 (67·1%) | ref | ref |  |
| Positive | 62 (22·5%) | 213 (77·5%) | 0·51 | 0·20, 1·37 | 71·3% |
| ***Opisthorchis viverrini* infection** |  |  |  |  |  |
| Negative | 76 (25·7%) | 220 (74·3%) | ref | ref |  |
| Positive | 11 (20·0%) | 44 (80·0%) | 0·66 | 0·28, 1·48 | 12·6% |
| ***Strongyloides stercoralis* infection** |  |  |  |  |  |
| Negative | 50/228 (21·9%) | 178/228 (78·1%) | ref | ref |  |
| Positive | 10/49 (20·4%) | 39/49 (79·6%) | 0·48 | 0·12, 1·62 | 60% |
| **Pemba Island** | n: 24 | n: 355 |  |  |  |
| **Age** |  |  |  |  |  |
| 5-11 years | 14 (7·6%) | 170 (92·4%) | ref | ref |  |
| 12-34 years | 9 (6·1%) | 139 (93·9%) | 0·90 | 0·36, 2·17 |  |
| 35-64 years | 1 (2·1%) | 46 (97·9%) | 0·54 | 0·05, 3·09 |  |
| **Sex** |  |  |  |  |  |
| Female | 12 (5·7%) | 198 (94·3%) | ref | ref |  |
| Male | 12 (7·1%) | 157 (92·9%) | 1·10 | 0·46, 2·65 |  |
| ***Trichuris trichiura* infection** |  |  |  |  |  |
| Negative | 0 (0·0%) | 55 (100·0%) | ref | ref |  |
| Positive | 24 (7·4%) | 300 (92·6%) | 14·39 | 1·32, 467·34 | 100% |
| ***Ascaris lumbricoides* infection** |  |  |  |  |  |
| Negative | 18 (6·2%) | 271 (93·8%) | ref | ref |  |
| Positive | 6 (6·7%) | 84 (93·3%) | 0·85 | 0·31, 2·06 | 25·0% |
| **Hookworm infection** |  |  |  |  |  |
| Negative | 22 (6·3%) | 325 (93·7%) | ref | ref |  |
| Positive | 2 (6·3%) | 30 (93·8%) | 0·75 | 0·13, 2·81 | 8·3% |

Abbreviations: CrI, credible interval; FC, fecal calprotectin; OR, odds ratio; ref, reference group

^a^ Adjusted for age, sex, *T. trichiura*, *A. lumbricoides* and hookworm infection status

^b^ Calculated as positive/(positive + negative) in the participants with FC ≥ 50 µg/mg

S2 Table. Determinants for fecal calprotectin levels in fecal stool samples – Country × *T. trichiura* infection model. Presented are odds ratios and 95% credible intervals estimated by multivariable logistic regression.^a^

| **Variable** | **OR** | **95% CrI** |
| --- | --- | --- |
| **Country ^b^** |  |  |
| Côte d’Ivoire | ref | ref |
| Lao PDR | 2·41 | 0·97, 6·15 |
| Pemba Island | 0·01 | 0·00, 0·16 |
| **Age categories** |  |  |
| 5-11 years | ref | ref |
| 12-34 years | 0·80 | 0·55, 1·17 |
| 35-64 years | 1·58 | 1·07, 2·35 |
| **Sex** |  |  |
| Female | ref | ref |
| Male | 1·11 | 0·81, 1·53 |
| ***Ttrichuris trichiura* infection ^b^** |  |  |
| Negative | ref | ref |
| Positive | 1·56 | 0·71, 3·49 |
| ***Ascaris lumbricoides* co-infection** |  |  |
| Negative | ref | ref |
| Positive | 1·19 | 0·86, 1·65 |
| **Hookworm co-infection** |  |  |
| Negative | ref | ref |
| Positive | 0·56 | 0·29, 1·01 |
| **Interactions** |  |  |
| Lao PDR × *T· trichiura* infection | 0·58 | 0·19, 1·77 |
| Pemba Island × *T· trichiura* infection | 30·04 | 1·56, 8038 |

Abbreviations: CI, credible interval; FC, fecal calprotectin; OR, odds ratio; ref, reference group

^a^ Regression model includes country, age, sex, *T. trichiura, A. lumbricoides,* hookworm infection status, and country × *T. trichiura* interaction term

**^b^** Main country and *T. trichiura* infection effects have a different interpretation in the presence of an interaction term
